# Clinical indications for plain abdominal radiographs: A survey study among radiographers

**DOI:** 10.4102/hsag.v28i0.2289

**Published:** 2023-08-29

**Authors:** Lynn Burrell, Razana Williams, Wilma ten Ham-Baloyi

**Affiliations:** 1 Department of Radiography, Faculty of Health Sciences, Nelson Mandela University, Gqeberha, South Africa; 2 Department of Nursing Science, Nelson Mandela University, Gqeberha, South Africa

**Keywords:** clinical indications, diagnostic radiography, radiographic examinations, abdominal radiographs, radiographers, knowledge, practices, radiographic image

## Abstract

**Background:**

Abdominal pain is a common complaint in the Emergency Department. Radiographers’ knowledge and practices regarding clinical indications for performing abdominal examinations are crucial in the results radiographs produced.

**Aim:**

To determine the knowledge and practices of radiographers regarding the clinical indications for performing radiographic examinations of the abdomen.

**Setting:**

Four public hospitals in the Eastern Cape province, South Africa.

**Methods:**

A cross-sectional study was conducted, using a convenience, all-inclusive sample of *n* = 85 radiographers. A hard copy self-administered questionnaire was distributed between February and June 2020. Descriptive (mean and standard deviations) and inferential (chi² test) statistics were generated using IBM^®^ SPSS^®^ version 26.0 software package.

**Results:**

Knowledge of clinical indications had a mean of 59.41. All four demographics (age, years of experience, attended a short course and attended pattern recognition course) were significantly associated with overall knowledge. Additionally, short course attendance was significantly associated with most practice items, and two knowledge items (which views are done for perforation; and which view(s) demonstrate a stab abdomen). Pattern recognition was significantly associated with one knowledge item (which views are regarded as an acute abdomen).

**Conclusion:**

Despite the lack of short courses and pattern recognition courses, radiographers’ knowledge of clinical indications was good (>50%). Continuous training, accessible protocols for performing clinical indications for plain abdominal radiographic examinations for radiographers, audit, feedback and reminders to enhance protocol adherence are recommended.

**Contribution:**

The study findings could be used to enhance knowledge and practices regarding clinical indications for plain abdominal radiographic examinations among radiographers.

## Introduction

Plain abdominal radiography is the initial imaging modality of choice when it comes to making a diagnosis for the abdomen (Tomizawa et al. 2017). It is defined as a means of obtaining imaging of the internal structures found in the abdominal cavity, by passing x-rays through them and recording the shadows cast by these structures (Lau 2007). Despite advancements in radiology such as computed radiography and ultrasound, plain abdominal radiography, using x-rays, still plays a significant role in the imaging protocol of the abdomen (Bell 2022). Patel, Moran and Nakada (2018) state that plain abdominal radiography is readily available and has the additional benefits of a lower radiation dose and lower cost factor to the patient.

Abdominal problems are common clinical complaints in the Emergency Department (ED) and account globally for 8% of all patient visits to the ED (Meltzer et al. 2017). These abdominal problems are wide and range from surgical to non-surgical conditions and from self-resolving to life-threatening conditions (Patterson, Kashyap & Dominique 2021; Velissaris et al. 2017). The clinical indications for abdominal radiographic examinations are normally grouped into suspected bowel obstruction, perforation, suspected foreign body, moderate to severe undifferentiated abdominal pain and renal tract calculi follow-up (Rink & Wessels 2021). However, plain imaging radiographs of the abdomen and chest are commonly requested for acute medical emergencies in patients who present with non-specific abdominal signs and symptoms (Fox 2017).

Gleadle, Li and Yong (2017) state that there are many diseases that could cause acute abdominal pain or acute abdomen and each of these has a specific imaging protocol to follow. Thus, the radiographer needs to have the necessary knowledge about the clinical indications as well as the protocols in order to assist in the diagnosis of the patient (European Society of Radiology 2019). Furthermore, the most appropriate abdominal protocol should be followed in order to demonstrate the pathology in question and, by applying the knowledge to practice, mismanagement and treatment delays are minimised, enhancing patient safety (Gleadle et al. 2017). It is important for the radiographer to follow the distinct protocol for each indication so that pathology is not overlooked. Radiographs allow the physician to see the pathology presented and when an incorrect protocol is followed, it leads to delay in treatment and risks that could include severe untreated pain, additional complications and even death of the patient, which compromises patient safety (Vom & Williams 2017). Furthermore, a protocol allows radiographers to accept a more autonomous role that cultivates critical thinking, reflection and research-informed decision making when performing or justifying performance of radiographic examinations (Vom & Williams 2017). Various protocols for abdominal radiography (indications) exist, including the Australian Diagnostic Pathways, The British Royal College, The American College of radiology, The French National Authority for Health and The Royal College of Radiologist Guidelines, which differ slightly from each other. Radiographers in South African public hospitals follow the provincial Department of Health’s protocol that lists the following indications: perforated abdomen, stab abdomen, subphrenic abscess and renal calculus (Department of Health 2010). However, as observed by the first author, there was evidence of radiographers in the study setting not adhering to this protocol by conducting inadequate projections when performing radiographic examinations of the abdomen. Non-adherence to protocols regarding plain abdominal examinations by radiographers could be a result of limited knowledge and practices, which ultimately impacts patients negatively thus affecting the safety and treatment plan of the patient (McFadden et al. 2018). Although studies have been conducted regarding the radiographers’ perspectives, knowledge and practices on x-ray examinations in Europe (McFadden et al. 2018; Wit, Vroonland & Bijwaard 2022), little is known on the knowledge and practices of radiographers with regard to the clinical indications for performing radiographic examinations of the abdomen, as evidenced by a paucity of articles in this regard found globally, including South Africa. This study, therefore, aims to determine the knowledge and practices of radiographers with regard to the clinical indications for performing radiographic examinations of the abdomen at public hospitals in the Eastern Cape, South Africa.

## Methods

This quantitative study used a cross-sectional survey design and was conducted by the first author under the supervision of the second and third authors. The study was part of a bigger study that was conducted to develop evidence-based recommendations regarding abdominal radiographic imaging practices for radiographers.

In the Eastern Cape, there are five radiology departments in five public hospitals, respectively. The main study was conducted at radiology departments in four public hospitals in the Eastern Cape, South Africa. The four hospitals were selected as they perform a high number of abdominal examinations. Radiographers employed in these hospitals have been trained in plain abdominal radiographs and the clinical indications for these and, while qualified, receive refresher short courses on performing abdominal examinations and pattern recognition as part of their continuous professional development.

To obtain as large as possible sample size, an all-inclusive, convenience sampling method was used to select the radiographers for the study.

### Survey questionnaire

The data collection instrument was a self-administered questionnaire, developed based on a literature review and aligned with the provincial Department of Health’s protocol (Department of Health 2010). Kindly note that this protocol may differ from other, international protocols. The questionnaire consisted of the following three sections:

- Section A – demographics, including age, years of experience, attendance at short courses and pattern recognition training (four questions).
- Section B – knowledge of radiographers with regard to clinical indications when performing abdominal radiographic examinations (eight multiple-choice questions).
- Section C – practices of radiographers with regard to the clinical indications when performing abdominal radiographic examinations (nine questions: five 5-point Likert Scale questions and four multiple-choice questions)

As part of the process to validate the questionnaire, a pilot test was conducted in a radiography department in one purposively selected hospital as this was the hospital with the lowest number of staff, consisting of 12 radiographers. The data obtained from the pilot study were used within the main study to enhance the sample size as no changes were made to the questionnaire after the pilot study was conducted.

After permission for the study was obtained, the managers at the hospitals (who acted as the gatekeepers) were informed of the study. After they agreed with the terms of the study, an information session at each of the hospitals was held during a weekly staff meeting so as to include all potential participants and not disrupt care. Participants were issued a consent form, which was signed and returned to the first author. Participants were then given a questionnaire which, once completed, was to be placed in a sealed box the researcher provided in the manager’s office and collected within 2 weeks. The manager sent a weekly reminder regarding completion of the questionnaires to participants.

To avoid contamination of data, participants were encouraged not to discuss the answers with each other. Similarly, they were encouraged not to use any other sources to inform their answers, including books or accessing information through electronic devices. Data were collected from April to June 2020. During data collection, COVID-19 protocols were adhered to and an agreement was made with the managers not to disrupt patient care during data collection.

### Data processing and statistical analysis

The completeness of the self-administered questionnaire was checked before capturing the data using IBM^®^SPSS^®^ version 26.0 software package. Descriptive (e.g. mean and standard deviation) and inferential (Chi² test) statistics were generated using IBM^®^ SPSS^®^ version 26.0 software package.3.0 The significance level was determined at *p* < 0.05. The scores for knowledge and practices were calculated as percentages, in the following manner:

Knowledge score = (Number of correct responses to knowledge items 1 to 8)/8 × 100.

An overall practice score could not be determined as there were no true and false responses, which are required to determine overall practice scores.

### Rigour

The data collection tool’s rigour was enhanced through the pilot study and review by the statistician and basing the questionnaire on existing protocol (Department of Health 2010). Additionally, the questionnaire was reviewed by an expert in radiography to ensure unleaded, simple, neutral questions were asked, and participants were assured the questionnaire was anonymous to mitigate performance and response bias.

Think about using anonymous surveys.

### Ethical considerations

Permission for the study was provided by the Faculty of Health Sciences at the relevant University (H19-HEA-RAD-008) and by the Provincial Department of Health (EC_201908_002). Permission was granted formally in writing from the CEOs at the different hospitals and verbally from the head of each radiology department. Informed consent was obtained from respondents, and the questionnaire was anonymous.

## Results

A total of 85 self-administered questionnaires were completed out of 118 questionnaires issued, resulting in a response rate of 72%. The demographic characteristics of the respondents are presented in Table 1.

**TABLE 1:** Demographic characteristics of the respondents (*N* = 85).

Demographics	Population size (*n*)	%
**Age (in years)**		
20–29	29	34.1
30–39	36	42.4
40–49	14	16.5
50–59	6	7.1
**Years of experience**		
1–4	14	16.5
5–9	31	36.5
10–15	26	30.5
≥ 16	13	15.3
No response	1	1.2
**Short course attendance (on performing abdominal examinations)**		
No	79	92.9
Yes	4	4.7
No response	2	2.4
**Pattern recognition training**		
No	73	85.9
Yes	12	14.1

%, percentage; n, number.

As outlined in Table 1, almost half (42.2%) of participants were found to be in the age category of 30–39 years of age and the majority (67.0%) had between 5 and 15 years of work experience. Most participants did not attend a short course (92.9%) nor any pattern recognition training (85.9%).

As the sample size of each of the four hospitals was small and unequal, it was not feasible for hospitals to be compared and thus the results concern all radiographers (*n* = 85) across the four hospitals.

### Knowledge and practices

Overall, the best-answered statement in terms of knowledge was B4, regarding which views are done for constipation, with a correct response rate of 100% (*n* = 85). The worst answered statement in the knowledge section was B1, regarding which views are done for diarrhoea, with a correct response rate of 1.2% (*n* = 1) (see Table 2).

**TABLE 2:** Knowledge and practices of clinical indications of the abdomen.

Variable	Correct		Incorrect	
	*n*	%	*n*	%
**Knowledge items**				
B1. Which view(s) are regarded as an acute abdomen (Answer D: Erect cxr,† erect axr‡ & supine axr)	74	87.1	11	12.9
B2. Which views will be done for renal pathology (Answer B: Erect & supine axr)	6	7.1	79	92.9
B3. Which views will be done for a perforation (Answer D: Erect axr & erect cxr)	72	84.7	13	15.3
B4. Which views will be done for constipation (Answer A: Supine axr)	85	100	0	0
B5. Which views will be done for diarrhoea (Answer B: Erect axr)	1	1.2	84	98.8
B6. Which views will be done for abdominal pain (Answer A: Acute axr series)	43	50.6	42	49.4
B7. Which views do you do for a stab chest (Answer C: Erect cxr)	73	85.9	12	14.1
B8. Which views will demonstrate stab abdomen (Answer D: Erect axr & erect cxr)	50	58.8	32	37.6
**Practice statements**				
C1. I always read the history of the patient before I do the requested examination (Agree)	80	94.1	4	4.7
C2. Patients must be referred back to the doctor if no history indicated (Agree)	75	88.3	9	10.6
C3. Doctors must be called in when forms are not filled in correctly (Agree)	74	87.1	10	11.8
C4. I perform additional views when pathology is noted (Agree)	77	90.6	6	7.1
C5. I follow incorrect protocols indicated on forms by doctors (Disagree)	76	89.4	8	9.3
C6. Abdominal views to do when a patient is unable to stand (decubitus axr C)	16	18.8	64	75.3
C7. When doctors request incorrect projections, do you (Do what department’s protocol says about listed indication & ignore doctors’ requests C)	51	60	32	37.4
C8. If a patient is stabbed left side of the abdomen, additional projections you would do (left decubitus A)	51	60	32	37.4
C9. Do you use or follow abdominal protocols afterhours (Yes B)	48	56.5	34	40

Note: Most participants had an average to high knowledge score (*n* = 71; 83.5%), with a mean knowledge score of 59.412, which was in the category of average to high (> 50).

%, percentage; *n*, number.

† , chest x-ray;

‡ , abdomen x-ray.

In terms of practices, question C1, stating ‘I always read the history of the patient before I do the requested examination’ received the highest number of correct responses at 94.1% (*n* = 80). Question C6, regarding abdominal views to do when a patient is unable to stand, received the lowest number of correct responses at 18.8% (*n* = 16) (see Table 2).

### Demographics and knowledge and practices

Chi-square tests were used to investigate the association between the variables such as age, years of experience, attendance at short courses, pattern recognition training and knowledge and practices (see Table 3, Table 4).

**TABLE 3:** Knowledge and practice items versus demographic items (*n* = 85).

Variable	Age			Years of experience			Short course attendance			Pattern recognition		
	Value	*df* †	*a* ‡	Value	*df*	*a*	Value	*df*	*a*	Value	*df*	*a*
**Knowledge items**												
B1. Which view(s) are regarded as an acute abdomen	2.443	6	0.927	6.694	8	0.422	7.792	4	0.201	17.520	4	0.041*
	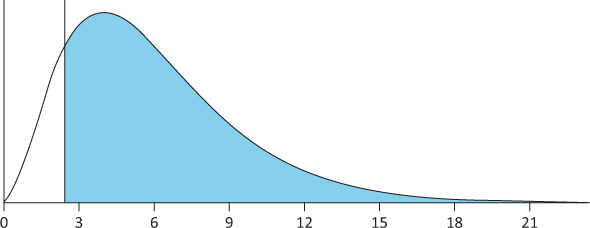	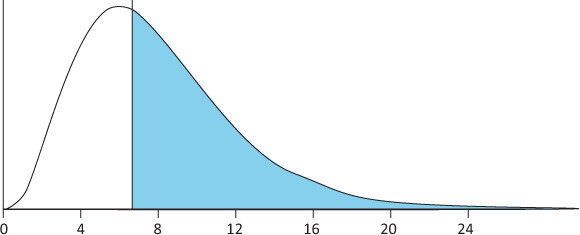	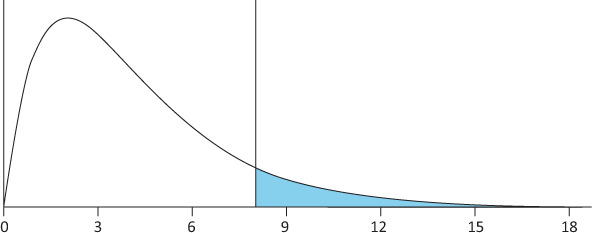	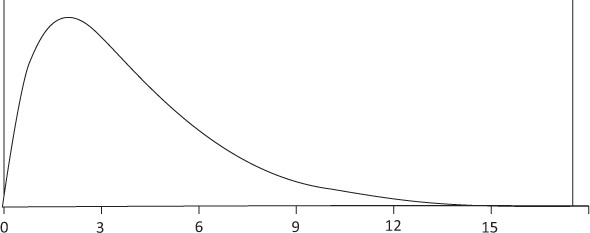
B2. Which views will be done for renal pathology	6.781	6	0.338	10.501	8	0.230	9.132	4	0.085	4.723	4	0.329
	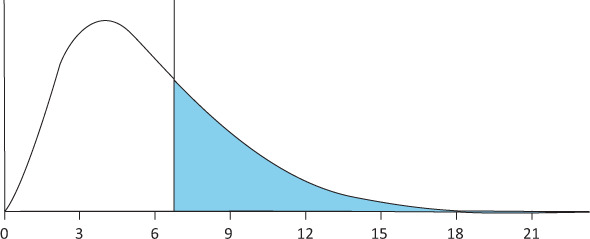	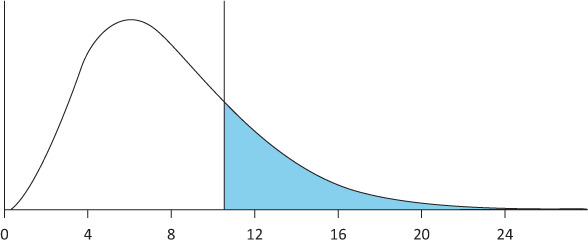	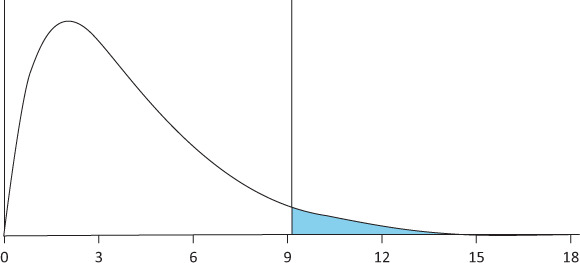	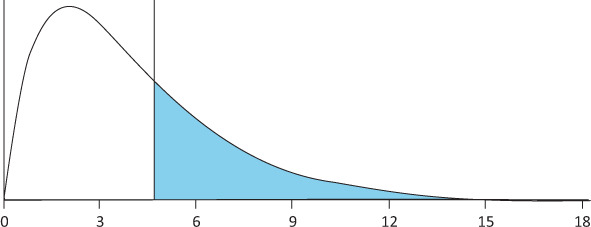
B3. Which views will be done for a perforation	2.368	6	0.912	6.600	8	0.506	46.468	4	0.002*	7.242	4	0.148
	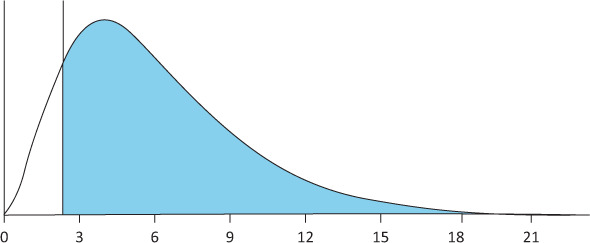	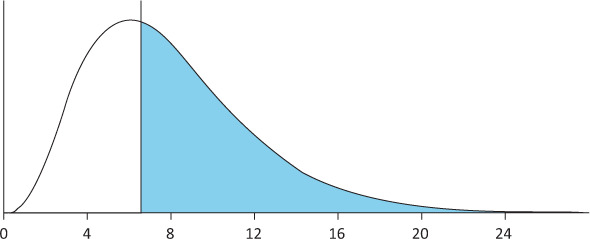	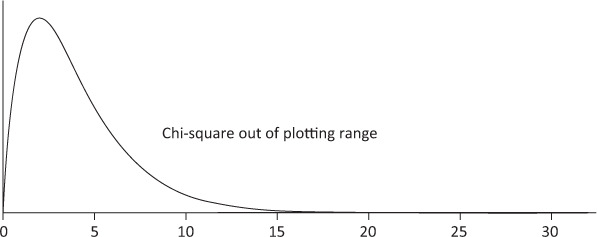	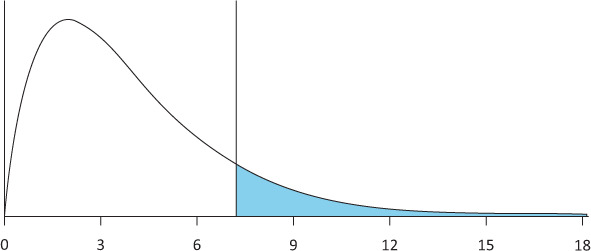
B4. Which views will be done for constipation	13.323	6	0.071	5.604	4	0.165	0.77	2	1.00	6.807	2	0.141
	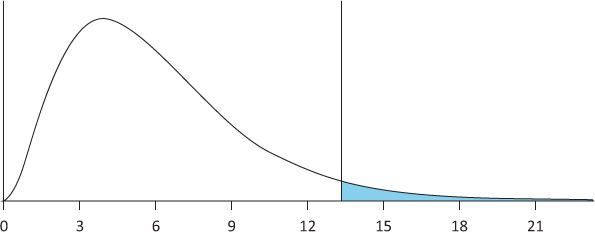	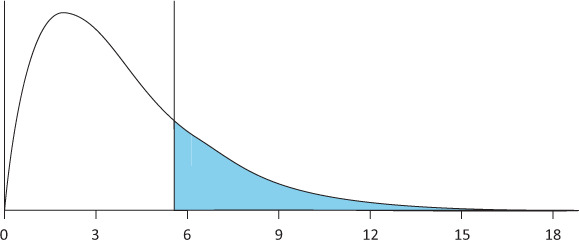	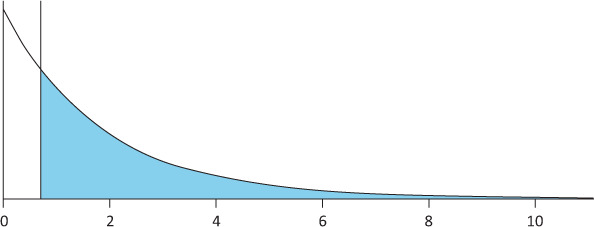	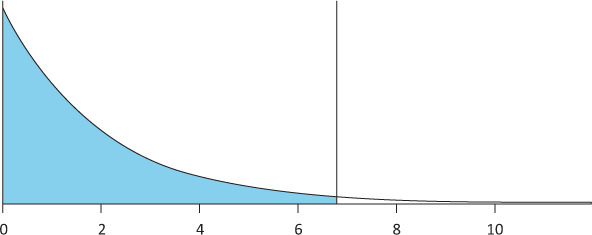
B5. Which views will be done for diarrhoea	10.808	9	0.275	9.445	12	0.627	4.132	6	0.437	1.145	6	0.947
	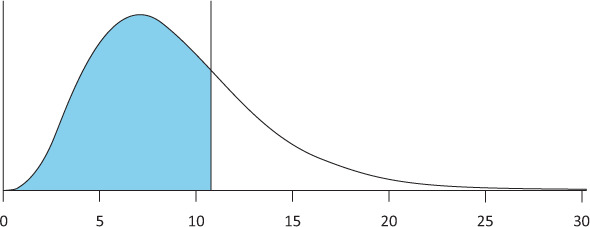	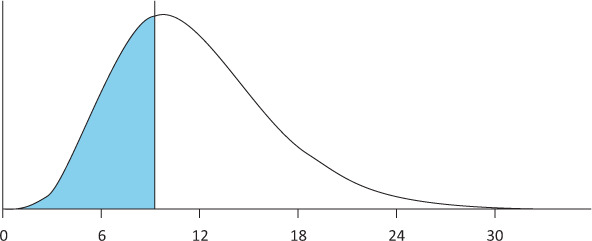	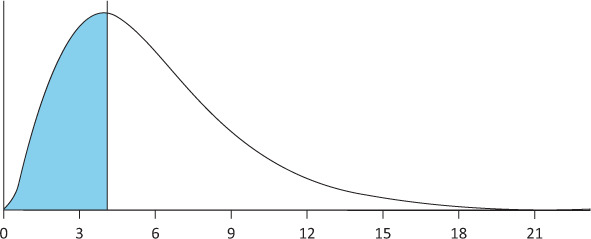	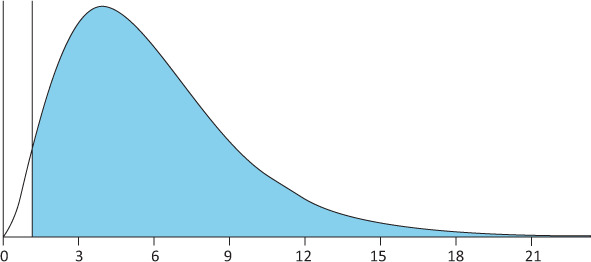
B6. Which views will be done for abdominal pain	8.407	9	0.504	8.704	12	0.687	5.146	6	0.428	5.223	6	0.365
	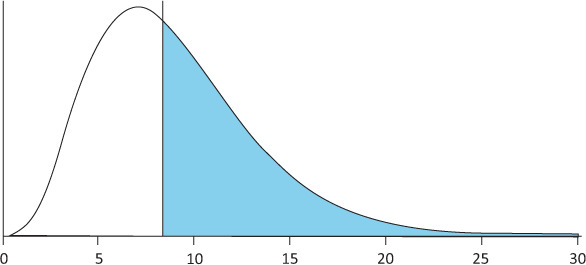	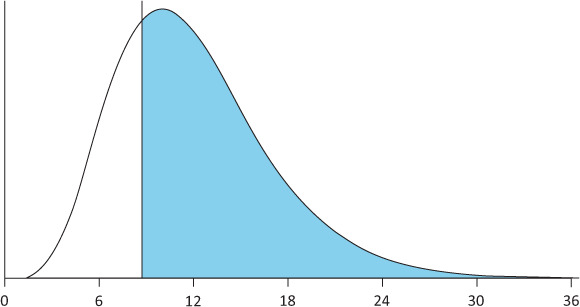	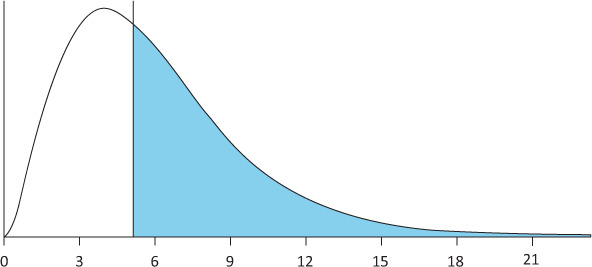	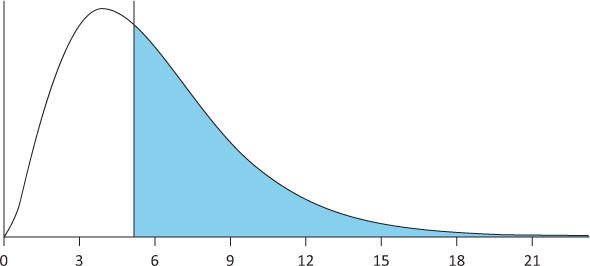
B7. Which views do you do for a stab chest	13.403	9	0.126	12.286	12	0.307	6.145	6	0.336	1.189	6	1.000
	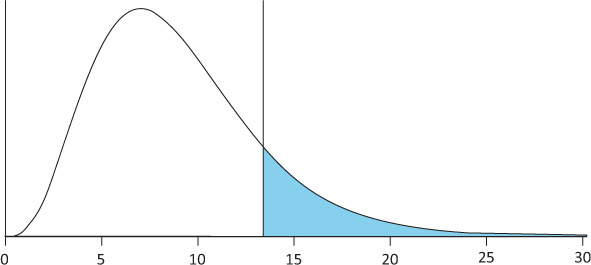	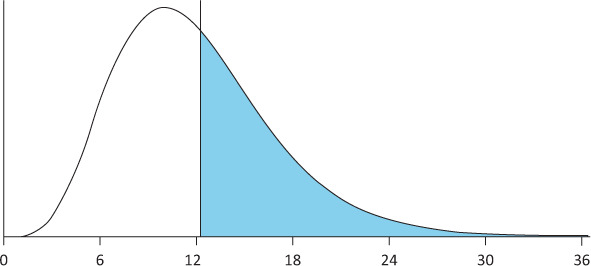	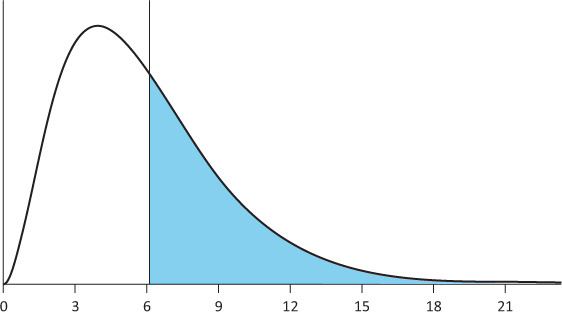	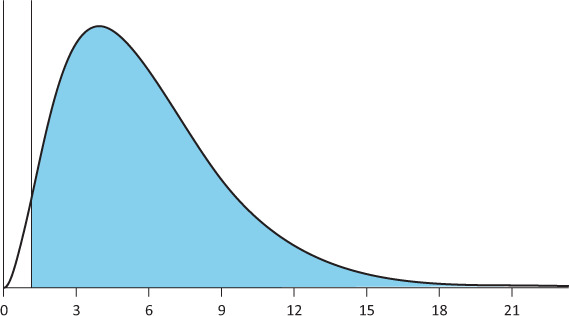
B8. Which views will demonstrate a stab abdomen	10.612	12	0.545	8.711	16	0.900	59.021	8	0.002*	2.203	8	0.831
**Pr actice statements**												
**C1.** I always read the history of the patient before I do the requested examination	9.634	12	0.608	15.395	16	0.335	46.671	16	0.049*	12.82	8	0.115
	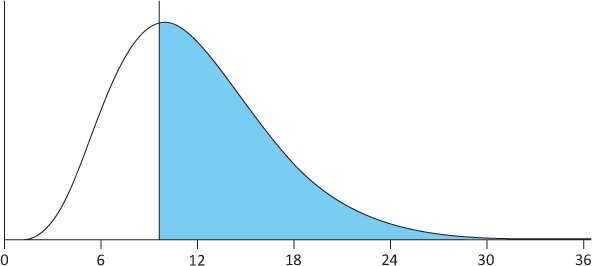	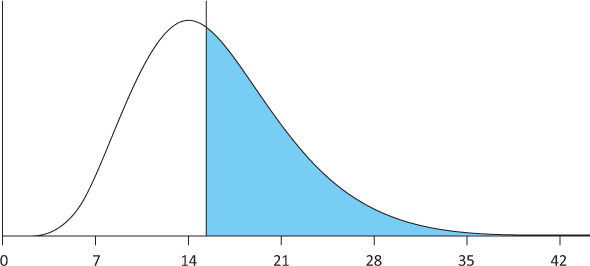	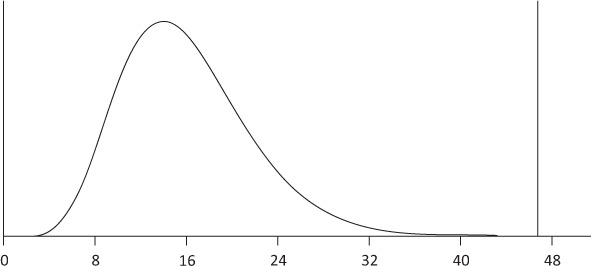	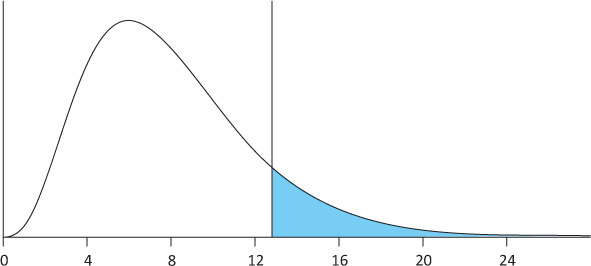
**C2.** Patients must be referred back to the doctor if no history is indicated	9.384	12	0.633	13.447	16	0.531	47.530	16	0.009*	12.271	8	0.121
	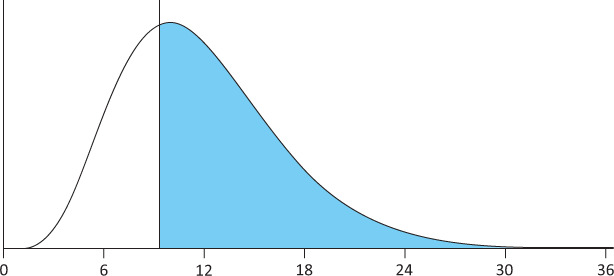	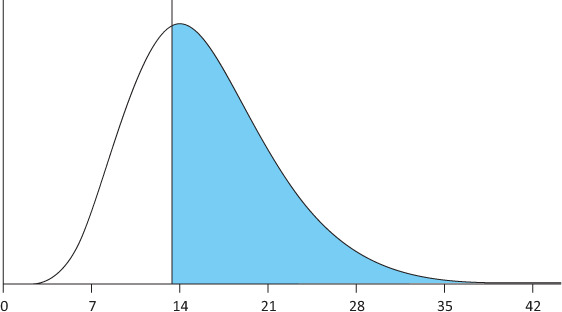	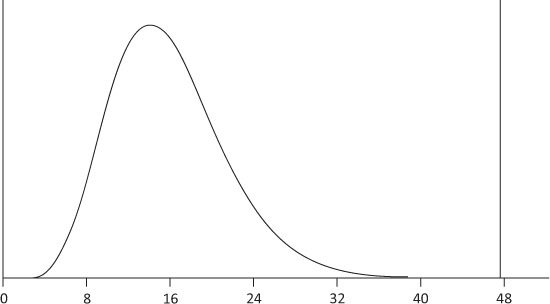	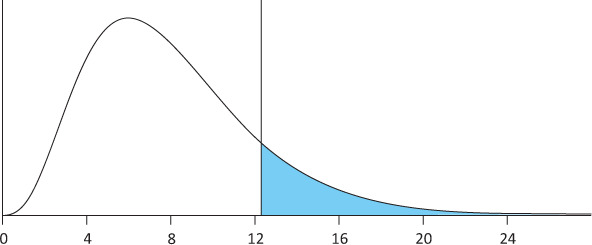
**C3.** Doctors must be called in when forms are not filled in correctly	14.114	12	0.283	15.991	16	0.340	64.983	16	0.001*	17.615	8	0.039*
	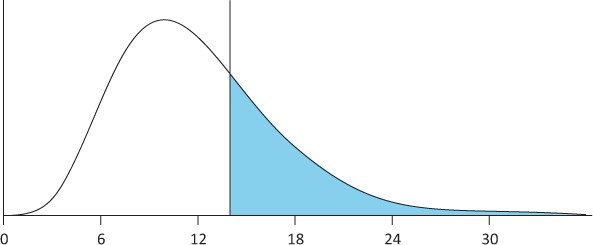	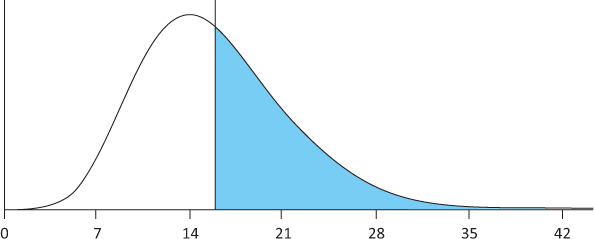	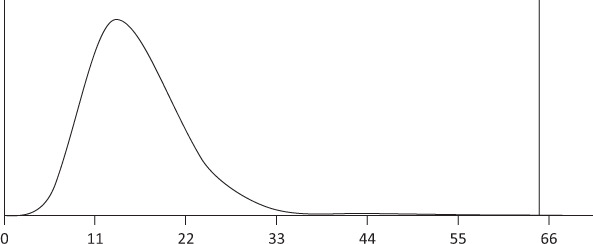	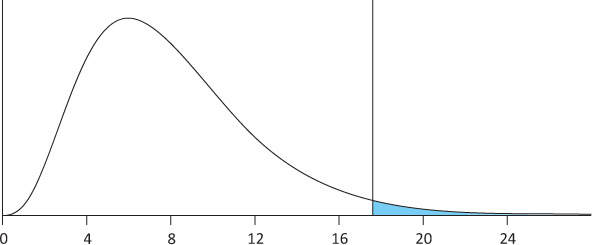
**C4.** I perform additional views when pathology is noted	5.306	12	0.961	12.470	16	0.585	21.383	16	0.108	5.681	8	0.387
	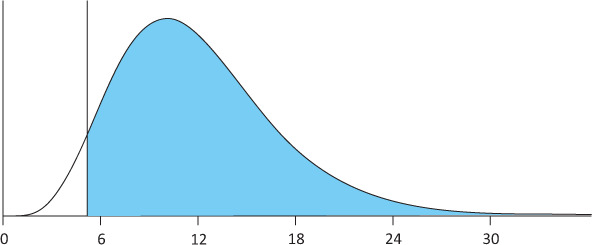	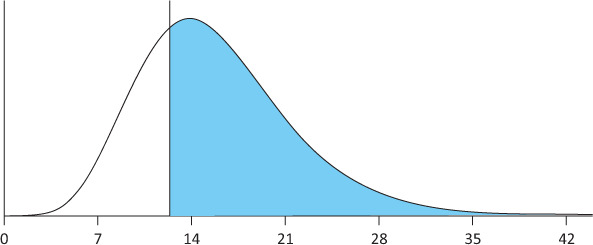	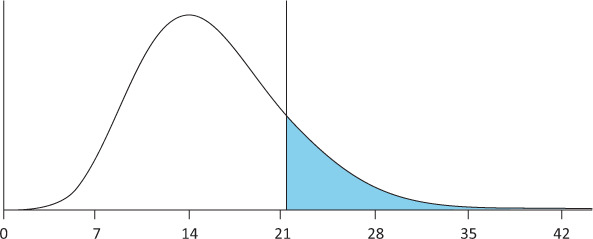	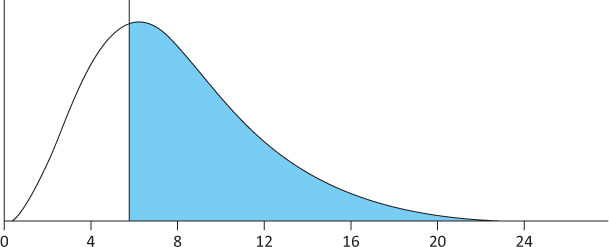
**C5.** I follow incorrect protocols indicated on forms by doctors	12.181	12	0.395	18.107	16	0.213	43.751	16	0.032*	95.094	8	0.001*
	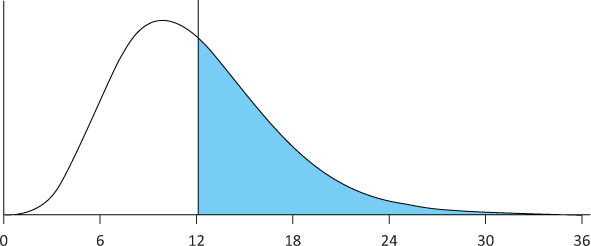	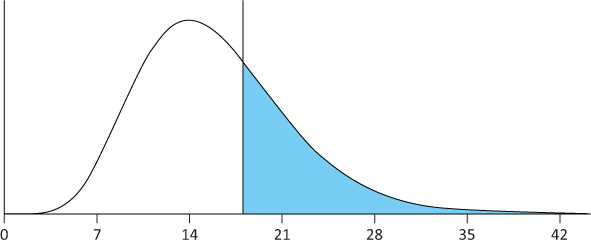	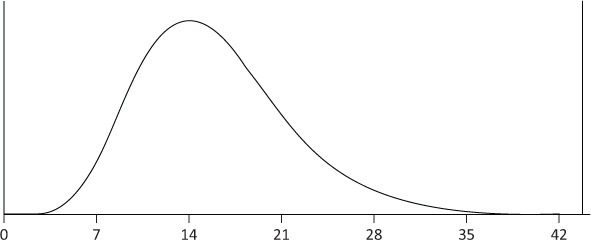	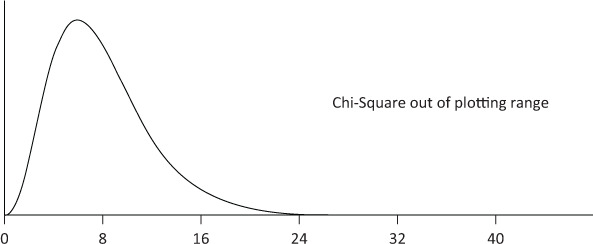
**C6.** Abdominal views to do when a patient is unable to stand	12.843	9	0.168	6.116	12	0.884	36.780	12	0.001*	2.292	6	0.788
	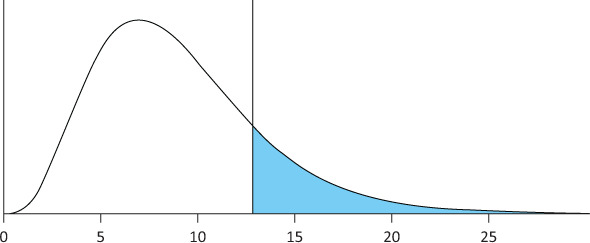	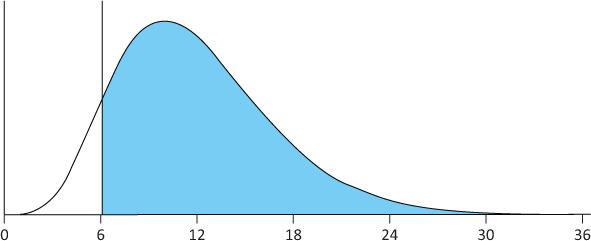	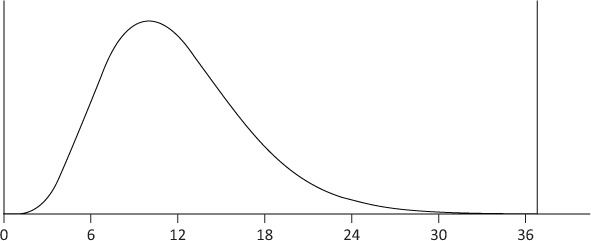	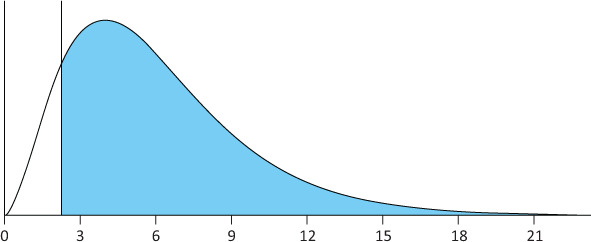
**C7.** When doctors request incorrect projections, what would you do?	5.252	9	0.829	9.349	12	0.602	85.566	12	0.000*	5.168	6	0.379
	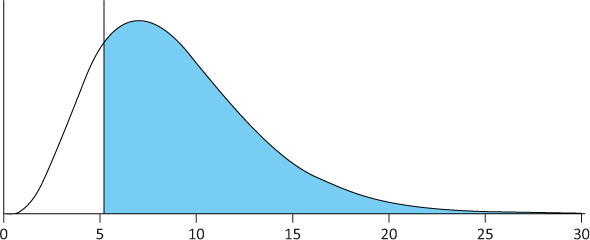	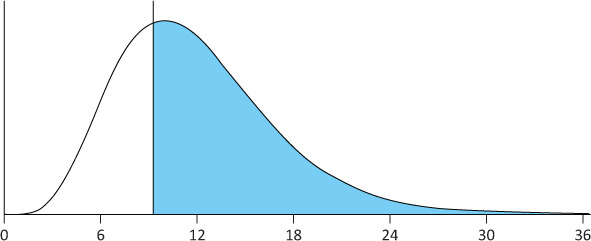	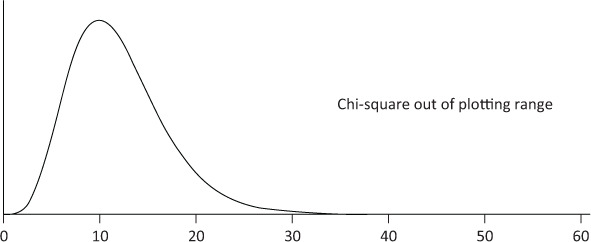	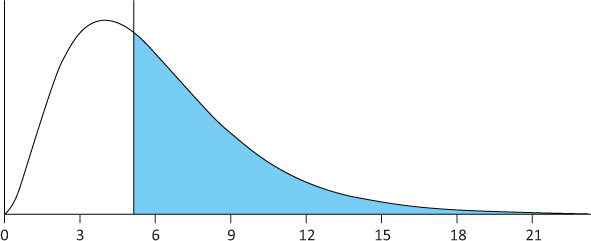
**C8.** If a patient is stabbed in the left side of the abdomen, additional views you would do	3.221	9	0.961	8.532	12	0.648	85.527	12	0.000*	12.659	6	0.082
	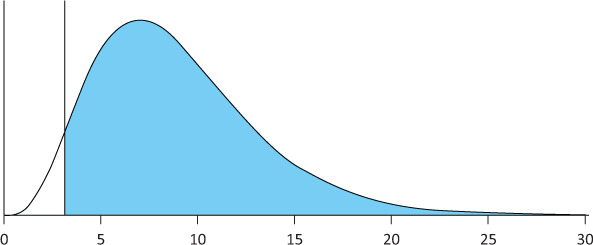	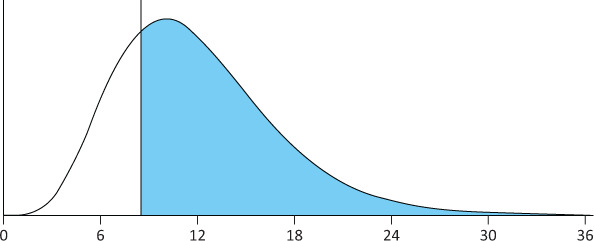	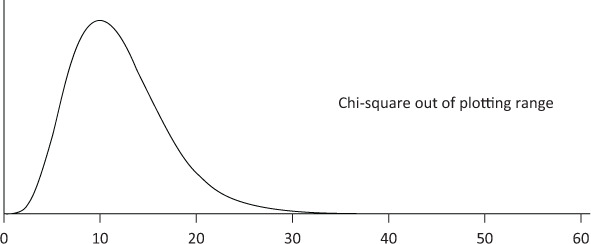	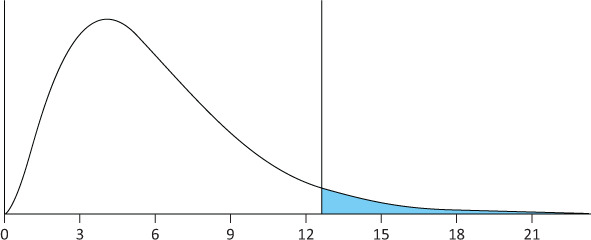
**C9.** Do you use/follow abdominal protocol after hours	9.029	9	0.438	9.977	12	0.491	56.529	12	0.001*	4.796	6	0.309
	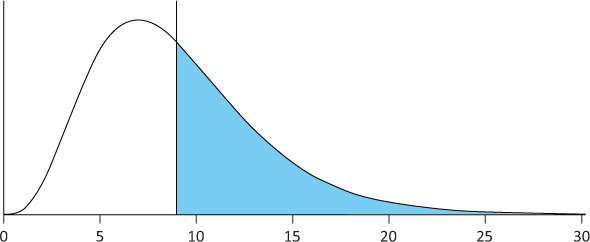	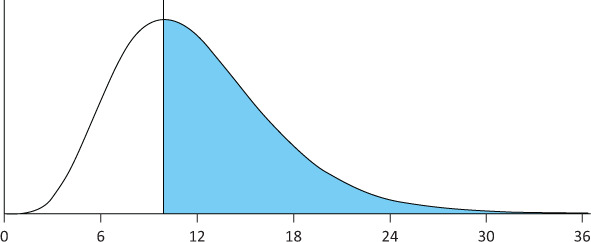	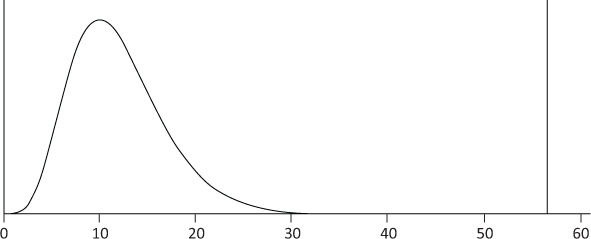	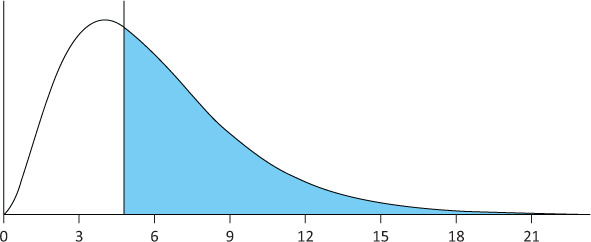

† , degree of freedom;

‡ , Significance (p < 0.05);

* , indicates significance.

**TABLE 4:** Overall knowledge score versus demographic items.

Demographic items	Type III Sum of squares	Degree of freedom (*df*)	Mean Square	*F*	Significance
Corrected model	6637.999	19	349.368	2.038	0.018*
Intercept	48523.604	1	48523.604	282.998	0.000*
Age	2608.104	3	869.368	5.070	0.003*
Years of experience	2816.847	3	938.949	5.476	0.002*
Short course	1083.374	2	541.687	3.159	0.049*
Pattern recognition	851.953	1	851.953	4.969	0.029*

* , indicates significance.

As outlined in Table 3, significant correlations were found between short course attendance and two of the eight knowledge items. These were B3, regarding which views are done for perforation (0.002), and B8, regarding which views demonstrate a stab abdomen (0.002). Similarly, significant correlations were found between short course attendance and eight of the nine practice items (C1, C2, C3, C5, C6, C7, C8 and C9). In addition, significant correlations were found between pattern recognition and one of the eight knowledge items, that is, B1 (Which view(s) are regarded as an acute abdomen) and two practice items: C3 and C5.

Table 4 outlines the overall knowledge score versus demographics items. As outlined, all four demographic items – namely, age (*p*-value is 0.003), years of experience (*p*-value is 0.002), short course (*p*-value is 0.049) and pattern recognition (*p*-value is 0.029) – were significantly correlated with the overall knowledge score.

## Discussion

This study aimed to determine the knowledge and practices of radiographers with regard to the clinical indications for performing radiographic examinations of the abdomen at public hospitals in the Eastern Cape. The study results revealed that although the majority of respondents indicated to have received minimal training in terms of short course attendance and pattern recognition related to abdominal radiographic examinations, overall knowledge of respondents regarding clinical indications for plain abdominal radiographic examinations can be described as above average. Most participants had an average to high knowledge score (*n* = 71; 83.5%), with a mean knowledge score of 59.412, which was in the category of average to high (> 50). Although average to high knowledge scores is generally regarded a good thing, there is scope for further increasing knowledge levels to enhance practice levels and subsequently service levels and improved patient outcomes (Lundvall, Dahlström & Dahlgren 2021). For example, enhanced knowledge of radiographers regarding clinical indications for plain abdominal radiographic examinations has been found to assist with optimisation of image quality and, subsequently, an accurate diagnosis and appropriate treatment (Alsleem et al. 2019).

The knowledge of respondents regarding clinical indications for performing radiographic examinations of the abdomen varied. The knowledge question regarding which view is done for constipation was answered the best, whereas which view is done for diarrhoea was answered worst, which could be contributed to the fact that the projection required for constipation is easier to obtain, namely, a supine view of the abdomen, which requires less moving and discomfort for a patient, as opposed to the erect radiograph considered as recommended practice for diarrhoea. The latter may therefore not be practised and, although a recommended practice, not remembered well, affecting knowledge scores (Geng et al. 2018). Furthermore, there seems to be a lack of adherence to protocols especially in the out of hours’ period, which would infer an inconsistent approach to imaging, and this could lead to health inequalities for patients presenting in the out of hours’ period to those during normal working hours. Also, ideally, all participants should have indicated that they always read clinical history as in order to justify the examination valid clinical indications must be included and read prior to exposure as it affects patient safety, as confirmed elsewhere (Ebben et al. 2018). Consistent adherences to protocols and recommended practices, crucial to equal and safe patient care can be enhanced through educational strategies, combined with audit and feedback as well as reminders to reinforce protocol adherence (Ebben et al. 2018).

Overall knowledge scores were significantly correlated with age, years of experience, short course attendance and pattern recognition training. Age as well as years of experience have been significantly associated with knowledge among radiographers elsewhere (Stami et al. 2018), and similar to this study could be attributed to the relatively young age of the respondents and medium years of experience (Ugwoke Eze, Uche Eze & Idogwu 2017). Furthermore, the importance of training – in this case attendance at short courses and pattern recognition training – is crucial to develop radiographers’ knowledge, professional skills and clinical competency, as recommended elsewhere (Tay & Kaur 2021). Additionally, possible knowledge gaps contributed by age and years of experience can be rectified in short courses and pattern recognition training (Moolman, Mulla & Mdletshe 2020). Attendance at short courses could enhance both knowledge and practices regarding abdominal examinations, as this study found these variables to be significantly correlated with the attendance of short courses, as found in a similar study by Farajollahi et al. (2014).

There is thus a need for sustained education and training regarding clinical indications for abdominal radiographic examinations among radiographers in this study. Additionally, it is recommended that all protocols on abdominal radiographic practices be available to radiographers. Regular in-service training should be provided regarding the protocols, as well as audits and feedback to enhance protocol adherence. Training on the protocols combined with protocol adherence strategies may result in consistently better knowledge and practices regarding clinical indications for plain abdominal radiographic examinations among radiographers, reducing inadequate visualisation of pathology that results in delayed treatment and misdiagnosis, enhancing patient safety and outcomes (Alsleem et al., 2019; Moolman et al. 2020).

This study was limited as the use of convenience sampling, the relatively small sample size and some of the knowledge items related to practices such as using erect AXR for diarrhoea and using acute AXR series for abdominal pain, which may not be common global practices, affects representativeness, hence limiting the generalisation of the study findings to a larger, global population. There is a need for further exploration of the reasons for the significant relationship between all demographic items and knowledge, which was not investigated in this study. Furthermore, although a pilot study was done to validate the questionnaire, questions and instructions could have been misunderstood by some respondents. Further adjustment and testing of the questionnaire are therefore recommended. Finally, as the study was limited to radiographers in public hospitals in the Eastern Cape only and random sampling because of the small population was not possible, the study should be repeated in radiology departments in public hospitals in South Africa.

## Conclusion

The results from this study revealed that although the majority of radiographers in radiology departments in four public hospitals in the Eastern Cape, South Africa, indicated to have received minimal training in terms of short course attendance and pattern recognition related to abdominal radiographic examinations, overall knowledge of respondents regarding clinical indications for plain abdominal radiographic examinations can be described as above average (*n* = 71; 83.5%), with a mean knowledge score of 59.412 (> 50). A significant association between all four demographics (age, years of experience, attended a short course and attended pattern recognition course) and overall knowledge was found. To enhance practices, there is a need for continuous training of radiographers regarding clinical indications for plain abdominal radiographic examinations based on protocols and guidelines, which should be made available to all radiographers as well as audits, feedback and reminders to enhance protocol adherence. Findings of this study could be used to obtain a better understanding of the level of knowledge and practices regarding clinical indications for plain abdominal radiographic examinations among radiographers. Recommendations to expand this study were provided and its findings could be used as the basis for the development of a national policy or strategic plan regarding clinical indications for plain abdominal radiographic examinations in radiology departments.
